# Full Separation or Full Integration? An Investigation of the Optimal Renewables Policy Employing Tradable Green Certificate Systems in Two Countries’ Electricity Markets

**DOI:** 10.3390/ijerph16244937

**Published:** 2019-12-05

**Authors:** Yanming Sun, Lin Zhang

**Affiliations:** 1 Institute for Global Innovation and Development, East China Normal University, 3663 North Zhongshan Rd., Shanghai 200062, China; ymsun@re.ecnu.edu.cn; 2 School of Urban and Regional Science, East China Normal University, Shanghai 200062, China; 3 Institute of Eco-Chongming, East China Normal University, Shanghai 200062, China; 4 School of Energy and Environment, City University of Hong Kong, Tat Chee Avenue, Kowloon, Hong Kong

**Keywords:** optimal renewables policy, tradable green certificates, full separation, full integration, welfare

## Abstract

Tradable green certificate (TGC) systems are increasingly used to promote renewable energy generation and mitigate greenhouse gas emissions. In this paper, we investigate the performance of the optimal renewables policy under full separation and full integration scenarios for two countries with TGCs. Our analysis suggests that under full separation, one country’s optimal renewable quota, which maximizes its own welfare, is strategically substitutional for the other country’s in a Cournot Nash equilibrium of the monopolistic market, when cross-border pollution exists. A country tends to become the “leader” in the market by using an information advantage to gain higher welfare. Using geometric illustrations we demonstrate the possibility that a potentially fully integrated electricity market under a TGC system can improve welfare for each country, when compensation between the countries is possible. From a policy point of view, this is significant in support of the demand for a convergence of national renewable policy schemes, where countries cooperate on solving cross-border environmental problems.

## 1. Introduction

Electricity generation from renewable sources experienced a boom in recent years with growing concerns on global climate change and environmental pollution. The development of renewable electricity will also lower the potential energy security risk associated with fossil fuels (in particular, oil) and create new economic opportunities and jobs. A set of policy instruments have been implemented by governments to encourage its growth [1,2,3]. Generally speaking, policy instruments aimed at promoting renewable electricity generation and mitigating greenhouse gas (GHG) emissions can be characterized along three dimensions. One is to either support investment in renewable energy production or subsidize renewable generation directly, for example, through feed-in tariffs (FIT). Another is to impose taxes on emissions or price emissions through a tradable permit system, such as the EU Emissions Trading Scheme (ETS). The third one is to regulate through a market-based system the “tradable green certificates” (TGCs) system. While the first two dimensions have garnered limited political support in both the United States and Canada, the third dimension—TGC systems—is now widely employed in a number of states in the U.S. (In the U.S., the TGC system is referred to as “renewable energy certificates” (RECs) or “renewable energy credits”), as well as in many other developed countries, including Australia, Japan, and most of the EU countries (these countries include the U.K., Denmark, Sweden, Norway, the Netherlands, Italy, Belgium, and Poland).

Under a TGC system, the final consumers and the distribution companies are obliged to ensure that a stipulated percentage of their electricity consumption is renewable. Renewable electricity generators are issued a green certificate for each unit of green electricity produced. Green certificates can be traded independently of electricity, and consumers can fulfill their obligation by purchasing certificates and handing them over to the authorities to prove their compliance. According to the literature, the TGC system has been shown to provide a cost-effective means of securing a certain proportion of renewables in the final consumption [4,5,6,7,8,9,10,11,12,13,14]. In the European Union, a fully harmonized support system, in the form of a pan-European quota obligation scheme with TGCs, was once concretely discussed, but ultimately was not implemented [8,9]. In 2009, the EU Renewables Directive stated that the EU member states may meet their national renewable targets by financing renewable energy production in other countries, and this so-called “statistical transfers” system can be seen as a first step towards a fully integrated green certificate system in the EU [2,10].

However, previous studies have given limited attention to countries’ strategic behavior within the context of electricity markets operated under TGCs in the presence of cross-border pollution. It is clear that energy market policy design in the context of countries’ cooperation in regulating cross-border pollution involves great challenges. Methodologically, most of studies in the literature treat renewable quota as a given target. Being different from them, this paper considers the renewable percentage requirement as a policy variable for the regulator to maximize welfare and promote renewables. Therefore, our modeling framework is capable of deriving the socially optimal level of renewable quota within the context of electricity market operated under the TGC system. By using geometric illustrations, we especially discuss each country’s percentage requirement reaction strategies in different competition types.

In this paper, based on the electricity duopoly in Currier et al. [15] and the Cournot electricity oligopoly framework by Currier and Sun [16], we construct a stylized theoretical model with numerical simulations to analyze the strategic competition behavior of two countries with cross-border environmental damages under full separation and full integration of their electricity markets and TGC markets. Under full separation, the two countries with the common border have their independent national electricity markets and green certificate markets. While the full integration situation is defined as when the two countries fully integrate their domestic electricity markets, they share a common renewable percentage requirement and a common green certificate market. Specifically, we compare several aspects of the performance of the optimal renewables policy under full integration and full separation, including welfare, environmental damage, imports/exports, and green/black output levels in each country. It demonstrates the possibility of achieving higher social welfare for each country through a common well-functioning TGC system than from fully independent national markets and TGC systems, as long as the possibility for compensation exists. 

Our results reveal that under full separation, the two countries’ optimal renewable percentage requirements are strategically substitutional in a Cournot–Nash equilibrium, and a country would always prefer to be the Stackelberg leader by using an information advantage. In addition, full integration between the two countries’ electricity markets with a common TGC system would be a Kaldor–Hicks improvement if the country made better off could compensate the country made worse off, as long as the possibility for compensation exists, but it does not necessarily make each country better off.

The remainder of this paper is organized as follows. Section 2 of the paper provides an overview of the literature and highlights our contributions. Section 3 presents the theoretical model. Section 4 fully analyzes each country’s percentage requirement reaction strategies for two types of competition under their full separation with common environmental damage. Section 5 tries to determine some implications of the impact of full integration by considering two scenarios. Section 6 concludes with a discussion.

## 2. Literature Review

This paper relates to three strings of literature. A group of studies have investigated countries’ cooperative/noncooperative behaviors in response to cross-border pollutions and the related consequences. For example, Hoel [17], Carraro and Siniscalco [18], and Barrett [19] suggested that the total emissions from all countries will not be much lower than they are in the noncooperative equilibrium. Following the foregoing, Hoel and Schneider [20] studied whether a system of side payments between countries reduces cooperation incentives or not, and both their theoretical analysis and numerical example suggested that transfers granted in international environmental agreements to free-riding countries sufficiently reduces the countries’ cooperation incentive and can result in higher total emissions. Böhringer and Rosendahl [21] studied the strategic partitioning of emission allowances by the EU member states between their trading and nontrading sectors. They examined the potential effects of the countries’ strategic behavior on emissions prices and abatement costs within the context of the EU emissions trading scheme. Tsakiris et al. [22] examined the fully cooperative, partially cooperative, and fully noncooperative trade and environmental tax policies based on a model of two large open economies with cross-border pollution. They also analyzed the case where both countries noncooperatively set their tax policies and examined the optimal response to their Nash pollution tax policies. Sun [23] discussed the optimal percentage requirement and welfare effects of a two-country electricity market with TGC system and found that full integration with a common TGC market is welfare superior to that of an entirely fossil fuel market with an optimal emissions standard. Helgesen and Tomasgard [14] studied the market power of TGC and welfare effects of a renewable power support scheme by formulating a multi-region partial equilibrium model, where they found that existing firms have to bear most of the deadweight losses from the policy. These studies have given limited attention to countries’ strategic behaviors within the context of TGCs in solving cross-border pollutions. In this study, we fully analyze the optimal renewables policy under the two bordering countries’ electricity markets with consideration of the cross-border pollution. 

Another group of studies have focused on the trends for countries that apply quota obligation schemes to integrate or converge their TGC systems. In Bye [6], a competitive electricity market model with a commitment by consumers to hold green certificates was presented. Their theoretical and numerical models yielded ambiguous price and volume effects for variations of green quota obligations, under both autarky and free trade of electricity and green certificates. Following the theoretical investigation of Amundsen and Mortensen [24], Amundsen and Nese [11] studied a Nordic-type TGC system in which integration occurred between Sweden and Norway. They especially analyzed how the system affects the generation of electricity from renewable sources and from carbon emitting sources, respectively. Widerberg [12] considered a domestic TGC system and an extended TGC system with trade. Distinguishing between the short run and long run, they discussed, for a situation in which a TGC system and an emissions trading scheme work together, how the change in the percentage requirement affects green and black electricity production. 

In this paper, by looking at two types of competition—the Cournot-type competition and the Stackelberg-type competition—we study the manner in which each country under regulation could strategically manipulate its renewable quota to maximize its own welfare. Using geometric illustrations, we clearly show each country’s percentage requirement reaction curves and iso-welfare curves and demonstrate that knowing the other country’s strategic behavior, a country would always prefer to be a Stackelberg leader in the game. 

The third group of literature is related to the rich improvement of various modeling approaches on alternative policy designs for renewable electricity within a country or among countries. Bushnell [25] studied how Cournot competitors may act strategically and increase profit by allocating more flexible hydro production to off-peak periods than they would under perfect competition. Based on a Cournot oligopoly with iso-elastic demand, Böhringer et al. [26] investigatd economic impacts from using feed-in tariffs or green certificates to promote renewable electricity within the EU. Vespucci et al. [27] represented the electricity market as a noncooperative game and assumed that generation firms are Cournot players that decide their strategy in order to maximize their profit, but their framework does not include support schemes for renewables. Gabriel et al. [28] solved Cournot–Nash energy production games while restricting some variables to be discrete in a recent power market study. Their approach allows for more realistic modeling but does not consider renewable support instruments. Nagl [29] looked at the effect of weather uncertainty on the financial risk of green electricity producers under feed-in tariffs and TGCs, where electricity demand is assumed to be inelastic. Tamas et al. [30] compared the performance of the feed-in and TGC systems in oligopolistic markets, where suppliers behave strategically under environmental regulations. Using the data from the UK market, they founnd that social welfare under TGC is consistently higher than FIT for a wide range of values of the parameters. Perez de Arce and Sauma [31] compared four incentive policies for renewable energy in an oligopolistic market with price-responsive demand. Ciarreta et al. [32] evaluate TGC and FIT as two alternative incentive schemes for renewable electricity. Their calibration with data from the Spanish electricity system suggests that as a regulatory system that reacts to market changes, the TGC system could both achieve the 2020 targets for renewables and reduce regulatory costs. Helgesen and Tomasgard [14] developed an equilibrium market model for electricity and TGCs under the Nash–Cournot competition. They showed that Cournot competition is a milder form of competition where firms are able to exploit market power. Existing firms will typically bear the biggest burdens from the TGC, and it may lead to substantial reallocations of welfare from existing firms to both consumers and new firms. Feng et al. [33] analyzed the dual effects of TGC and carbon emissions trading (CET) on the electricity market in China based on system dynamics models, where the simulation results showed advantageous impacts of renewable portfolio standards and carbon price on the optimization of power supply structure. Aune et al. [10] explored the impact of full trade in green certificates across the EU member states by evaluating different policy scenarios in a competitive situation. They found that cost effectiveness can be achieved by imposing a common renewable target for all countries and allowing for free trade in green certificates, whereas differentiated national targets cannot ensure a cost effective implementation to reach the overall renewable target. 

Many of the above studies treat the renewable quota obligation as a given parameter or a target to achieve, without considering the cross-border environmental damage from fossil energy production. They represent cost-effectiveness in achieving the renewable target as one important aspect in evaluating policy schemes. In contrast to them, this study provides a perspective of the TGC market designs between countries. We treat the renewable percentage requirement as a policy variable and as the only instrument that the regulator has to promote renewables and reduce GHG emissions. Thus, we are able to look at the socially optimal renewable quota within the context of electricity markets operated under TGC systems. In addition, we consider the common environmental damage when evaluating welfare and when analyzing each country’s percentage requirement reaction strategies under full separation. This gives more structure to the model when analyzing various types of competition. In fact, analyzing the welfare impact is especially interesting from a policy perspective. It broadly evaluates consumers’ utilities, production costs, environmental damages, and so forth and provides a clearer picture of what is driving the main results. 

## 3. The Methodological Framework

We consider a situation where two countries share a common border so that imports/exports of electricity are possible between them. It is assumed that the electricity trade does not affect the terms of trade. For simplicity of analysis, we do not consider CO_2_ taxes or other energy taxes and also ignore the trade of electricity with third countries. Each country’s energy market is served by two kinds of firms: fossil fuel producers of black electricity, $y_{i}$, and renewable producers of green electricity, $x_{i},\;i=1,\;2$. The cost functions for green and black output are $C_{ix}\left(x_{i}\right)$ and $C_{iy}\left(y_{i}\right)$, respectively, satisfying $C_{ix}^{\prime }\left(x_{i}\right)>0$, $C_{ix}^{\prime \prime }\left(x_{i}\right)>0$, $C_{iy}^{\prime }\left(y_{i}\right)>0$, $C_{iy}^{\prime \prime }\left(y_{i}\right)>0$, i=1, 2. We assume that there are no transportation costs. Let $z_{1}$ and $z_{2}$ denote the electricity consumption in country 1 and country 2, respectively. Consumer spending on electricity is not sufficiently important, so the income effect of demand can be ignored. $U_{i}\left(z_{i}\right)$ gives the consumer’s utility in country i, which is twice continuously differentiable with $U_{i}^{\prime }>0$ and $U_{i}^{\prime \prime }<0$, i=1,2.

### 3.1. Full Separation of Two Bordering Countries’ Electricity Markets

Under full separation, the two countries with the common border have their national electricity markets and green certificate markets. In each country’s green certificate market with consumer obligations to hold green certificates, every consumer of electricity is obliged to purchase $\alpha_{i}$ green certificates for each unit of electricity they consume, $\alpha_{i}\in [0,1]$, i=1,2. So, the actual consumer price is $p_{i}+p_{c_{i}}\alpha_{i}$, where $p_{i}$ denotes the market price of electricity in country i, and $p_{c_{i}}$ denotes the unit price of green certificates in country i. The domestic demand for electricity is given by the maximization of the consumer surplus, which is formed by:

(1) $$Max\;CS_{i}=U_{i}(z_{i})-(p_{i}+p_{c_{i}}\alpha_{i})z_{i},\;i=1,2$$

Each green producer in country i sells one green certificate for every unit of green electricity being sold out, so they will receive $p_{c_{i}}$ for each unit in addition to the price of electricity, i=1,2. Hence, the green producers’ profits in country i are: $\pi_{ix}=(p_{i}+p_{c_{i}})x_{i}-C_{ix}(x_{i}),\;i=1,2$. The black producers’ profits in country i are: $\pi_{iy}=p_{i}y_{i}-C_{iy}(y_{i}),\;i=1,2$. When the domestic electricity market clears, $x_{i}+y_{i}=z_{i},\;i=1,2$. When the TGC market in country i clears, $x_{i}=\alpha z_{i},\;i=1,2$, and thus, the renewable quota is satisfied. We assume throughout that the constraint is binding and is thus satisfied as an equality in equilibrium.

Considering the cross-border pollution between these two countries, let $y=y_{1}+y_{2}$ and $D_{i}(y_{1}+y_{2})$ denote the environmental damage in country i caused by fossil energy production in these two countries, i=1,2. Assume that $D_{i}^{\prime }>0$ and $D_{i}^{\prime \prime }\geq 0$. The social welfare in country i, i=1,2, is defined as $W_{i}=CS_{i}+\pi_{ix}+\pi_{iy}-D_{i}(y_{1}+y_{2})$, which is the unweighted sum of the consumer surplus and the industry profits net of the environmental damages in country i. When the domestic electricity market and green certificate market both clear, $W_{i}=U_{i}(z_{i})-C_{ix}(x_{i})-C_{iy}(y_{i})-D_{i}(y_{1}+y_{2})$, i=1,2.

With the competitive price-taking behavior in each country’s electricity market, the representative renewable and fossil-fuel producers maximize profits by choosing their output levels, which satisfy:

(2) $$C_{ix}^{\prime }(x_{i})-p_{c_{i}}=p_{i},\;i=1,2$$

(3) $$C_{iy}^{\prime }(y_{i})=p_{i},i=1,2$$

Consumers maximize the consumer surplus by choosing their consumption quantities, which satisfy:

(4) $$U_{i}^{\prime }-p_{c_{i}}\alpha_{i}=p_{i},i=1,2$$

Equations (2)–(4) give us the relationship that $U_{i}^{\prime }-p_{c_{i}}\alpha_{i}=C_{ix}^{\prime }(x_{i})-p_{c_{i}}=C_{iy}^{\prime }(y_{i})=p_{i}$, i=1,2. Thus, the demand function for electricity in country i can be written as $z_{i}(p_{i}+p_{c_{i}}\alpha_{i})$, i=1,2, and the supply functions for green electricity and black electricity are $x_{i}(p_{i}+p_{c_{i}})$ and $y_{i}(p_{i})$, i=1,2, respectively. For each value of $\alpha_{i}\in [0,1]$, there will be the unique competitive equilibrium (CE) electricity price ($p_{i}(\alpha_{i})$) and green certificate price ($p_{c_{i}}(\alpha_{i})$), implying equilibrium output levels $x_{i}^{CE}(\alpha_{i})$, $y_{i}^{CE}(\alpha_{i})$ and the equilibrium demand level $z_{i}^{CE}(\alpha_{i})$.

Recall that when the domestic TGC market and the electricity market both clear, the social welfare ($W_{i}$) for country i is $W_{i}=U_{i}(z_{i})-C_{ix}(x_{i})-C_{iy}(y_{i})-D_{i}(y_{1}+y_{2})$. In the competitive equilibrium, for each value of $\alpha_{i}\in [0,1]$,

$W_{i}=U_{i}(z_{i}(\alpha_{i}))-C_{ix}(x_{i}(\alpha_{i}))-C_{iy}(y_{i}(\alpha_{i}))-D_{i}(y_{1}(\alpha_{1})+y_{2}(\alpha_{2}))$. $W_{i}$ is a strictly concave function of $\alpha_{1}$ and $\alpha_{2}$. 

Let us define $S_{i}(\alpha_{i})=U_{i}(z_{i}(\alpha_{i}))-C_{ix}(x_{i}(\alpha_{i}))-C_{iy}(y_{i}(\alpha_{i}))$, i=1,2, and then the social welfare for country 1 and country 2 are $W_{1}(\alpha_{1},\alpha_{2})=S_{1}(\alpha_{1})-D_{1}(y_{1}(\alpha_{1})+y_{2}(\alpha_{2}))$ and $W_{2}(\alpha_{1},\alpha_{2})=S_{2}(\alpha_{2})-D_{2}(y_{1}(\alpha_{1})+y_{2}(\alpha_{2}))$, respectively.

### 3.2. Full Integration for Two Bordering Countries’ Electricity Markets

When the two countries with the common border have their domestic electricity markets and TGC markets fully integrated, they share a common renewable percentage requirement, α∈[0,1], and a common green certificate market. We assume there is free trade and no transportation costs between these two countries. Then, the actual consumer price is $p+p_{c}\alpha$, where p denotes the market price of electricity, and $p_{c}$ denotes the unit price of green certificates. The domestic demand for electricity is given by the maximization of the consumer surplus, which is formed by:

(5) $$Max\;CS_{i}=U_{i}(z_{i})-(p+p_{c}\alpha )z_{i},i=1,2$$

The green producers’ profits in country i are $\pi_{ix}=(p+p_{c})x_{i}-C_{ix}(x_{i}),\;i=1,2$. The black producers’ profits in country i are $\pi_{iy}=py_{i}-C_{iy}(y_{i}),\;i=1,2$. When the integrated electricity market goes to equilibrium, $x_{1}+x_{2}+y_{1}+y_{2}=z_{1}+z_{2}$. When the TGC market clears, the renewable quota is satisfied by $x_{1}+x_{2}=\alpha (z_{1}+z_{2})$. We still assume throughout that the constraint is binding and is thus satisfied as an equality in equilibrium. With the cross-border pollution between these two countries, $D_{i}(y_{1}+y_{2})$, i=1,2. 

The social welfare in country i is $W_{i}=CS_{i}+\pi_{ix}+\pi_{iy}-D_{i}(y_{1}+y_{2})$, i=1,2. When the fully integrated electricity market and green certificate market both clear, $W_{i}=U_{i}\left(z_{i}\right)-p\left(z_{i}-x_{i}-y_{i}\right)\;-p_{c}\left(\alpha z_{i}-x_{i}\right)-C_{ix}\left(x_{i}\right)-C_{iy}\left(y_{i}\right)-D_{i}\left(y_{1}+y_{2}\right),\;i=1,\;2$.

With the competitive price-taking behavior in the electricity market, the profits of green and black producers are maximized by choosing their output levels, which satisfy:

(6) $$C_{ix}^{\prime }(x_{i})-p_{c}=p,\;i=1,2$$

(7) $$C_{iy}^{\prime }(y_{i})=p,i=1,2$$

Consumers maximize the consumer surplus by choosing their consumption quantities, which satisfy:

(8) $$U_{i}^{\prime }-p_{c}\alpha =p,i=1,2$$

Equations (6)–(8) give us the relationship that $U_{i}^{\prime }-p_{c}\alpha =C_{ix}^{\prime }(x_{i})-p_{c}=C_{iy}^{\prime }(y_{i})=p$, i=1,2. Thus, the demand function for electricity in country i can be written as $z_{i}(p+p_{c}\alpha )$, i=1,2, and the supply functions for green electricity and black electricity are $x_{i}(p+p_{c})$ and $y_{i}(p)$, i=1,2, respectively. 

For each value of α∈[0,1], there will be the unique competitive equilibrium (CE) electricity price (p(α)) and green certificate price ($p_{c}(\alpha )$), implying equilibrium output levels $x_{i}^{CE}(\alpha )$, $y_{i}^{CE}(\alpha )$ and the equilibrium demand level $z_{i}^{CE}(\alpha )$. In the competitive equilibrium, for each value of α∈[0,1]:

$W_{i}=U_{i}(z_{i}(\alpha ))-p(z_{i}(\alpha )-x_{i}(\alpha )-y_{i}(\alpha ))-p_{c}(\alpha z_{i}(\alpha )-x_{i}(\alpha ))\;-D_{i}(y_{1}(\alpha_{1})+y_{2}(\alpha_{2}))$.

## 4. Alternative Competition Types under Full Separation

The electricity market has been traditionally recognized as fairly concentrated [29]. However, perfect competition is probably the ultimate goal after years of deregulation and liberalization of energy markets. In this study, for the simplicity of our analysis, we assume an ideal situation that the market in each country is competitive and allow countries to make strategic decisions.

As noted earlier, we shall fully analyze the optimal renewable policies for the two countries that share the common border with fully separated electricity markets and green certificate markets. We especially focus on the manner in which each country under regulation could strategically manipulate its renewable quota to maximize the country’s own welfare. In particular, when we turn our attention to the challenge of regulating a polluting, oligopolistic industry where the two countries serve as players in the game, the Cournot model and the Stackelberg model are useful starting points for understanding countries’ strategic behaviors. Therefore, we follow a traditional analytical framework by considering these two modeling approaches where countries compete in quota obligation to proceed our analysis.

### 4.1. Cournot-Type Competition

#### 4.1.1. Percentage Requirement Reaction Curves

Under full separation, we assume the renewable percentage requirement is the only policy instrument that the regulator has to reduce emissions and promote renewable production. Thus, each country’s renewable quota is a strategic choice variable. In this game, the two countries act simultaneously as Cournot players when choosing their national renewable quota obligations. Now, the regulator in country 1 wants to select $\alpha_{1}$ to maximize country 1’s social welfare. If we take the first-order condition of $W_{1}(\alpha_{1},\alpha_{2})$ with respect to $\alpha_{1}$, we can get:

(9) $$S_{1}^{\prime }(\alpha_{1})=D_{1}^{\prime }(y_{1}(\alpha_{1})+y_{2}(\alpha_{2}))\cdot y_{1}^{\prime }(\alpha_{1})$$

The second-order condition of $W_{1}(\alpha_{1},\alpha_{2})$ with respect to $\alpha_{1}$ gives:

(10) $$S_{1}^{\prime \prime }(\alpha_{1})-D_{1}^{\prime \prime }(y_{1}(\alpha_{1})+y_{2}(\alpha_{2}))\cdot {\left(y_{1}^{\prime }(\alpha_{1})\right)}^{2}-D_{1}^{\prime }(y_{1}(\alpha_{1})+y_{2}(\alpha_{2}))\cdot y_{1}^{\prime \prime }(\alpha_{1})<0$$

Equation (9) yields the optimal percentage requirement ($\alpha_{1}^{*}$) that maximizes $W_{1}$, where $\alpha_{1}^{*}=\alpha_{1}^{*}(\alpha_{2})$. So, when $\alpha_{1}$ takes the optimal value, say $\alpha_{1}^{*}$, Equation (9) can be written as $S_{1}^{\prime }(\alpha_{1}^{*}(\alpha_{2}))=D_{1}^{\prime }(y_{1}(\alpha_{1}^{*}(\alpha_{2}))+y_{2}(\alpha_{2}))\cdot y_{1}^{\prime }(\alpha_{1}^{*}(\alpha_{2}))$. If we differentiate this with respect to $\alpha_{2}$ and solve for $\frac{d\alpha_{1}^{*}}{d\alpha_{2}}$, we can show that:

(11) $$\frac{d\alpha_{1}^{*}}{d\alpha_{2}}=\frac{D_{1}^{\prime \prime }\cdot \frac{\partial y_{2}}{\partial \alpha_{2}}\cdot y_{1}^{\prime }(\alpha_{1}^{*}(\alpha_{2}))}{\left[S_{1}^{\prime \prime }(\alpha_{1}^{*})-D_{1}^{\prime \prime }\cdot {\left(y_{1}^{\prime }(\alpha_{1}^{*}(\alpha_{2}))\right)}^{2}-D_{1}^{\prime }\cdot y^{\prime \prime }(\alpha_{1}^{*}(\alpha_{2}))\right]}$$

In Equation (11), $D_{1}^{\prime \prime }\geq 0$, $\frac{\partial y_{2}}{\partial \alpha_{2}}<0$ and $y_{1}^{\prime }(\alpha_{1}^{*}(\alpha_{2}))<0$, so the numerator is positive. From Equation (10), the denominator of $\frac{d\alpha_{1}^{*}}{d\alpha_{2}}$ is negative. Thus, $\frac{d\alpha_{1}^{*}}{d\alpha_{2}}\leq 0$, and when $D_{1}^{\prime \prime }=0$, $\frac{d\alpha_{1}^{*}}{d\alpha_{2}}=0$. Similarly, $\frac{d\alpha_{2}^{*}}{d\alpha_{1}}\leq 0$, and when $D_{2}^{\prime \prime }=0$, $\frac{d\alpha_{2}^{*}}{d\alpha_{1}}=0$.

**Proposition** **1.** In a Cournot-type competition, in choosing the renewable quota obligations under full separation, the optimal renewable percentage requirement in one country is a reaction function of the other country’s. In a Cournot–Nash equilibrium, $\frac{d\alpha_{1}^{*}}{d\alpha_{2}}\leq 0$*,*$\frac{d\alpha_{2}^{*}}{d\alpha_{1}}\leq 0$, which means the two countries’ percentage requirements are strategic substitutes, considering cross-border pollution. That is, as one country increases its green quota, the other country will strategically reduce its renewable quota in response, in order to maximize its own welfare.

**Figure 1:**
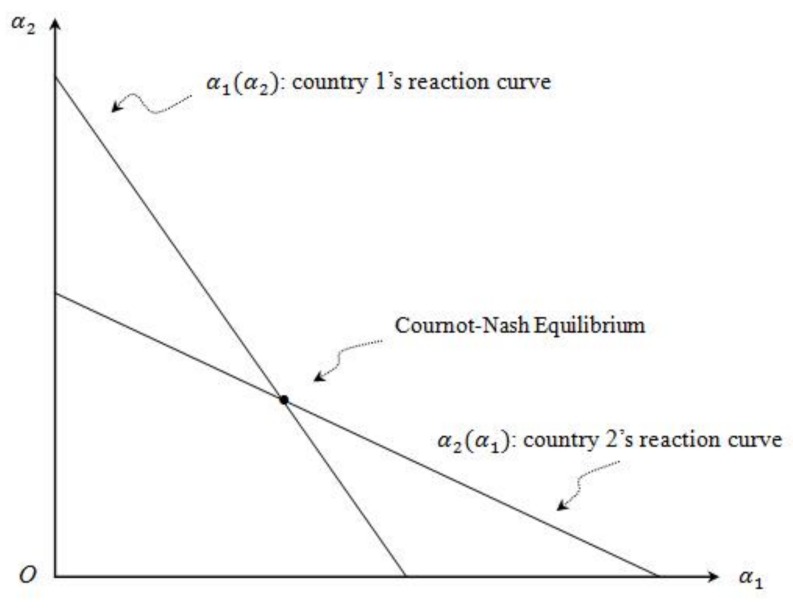
The reaction curve for each country.

We do not consider the special cases that $\frac{d\alpha_{1}^{*}}{d\alpha_{2}}=0$ and $\frac{d\alpha_{2}^{*}}{d\alpha_{1}}=0$ when $D_{i}^{\prime \prime }=0$, i=1,2. With $\frac{d\alpha_{1}^{*}}{d\alpha_{2}}<0$ and $\frac{d\alpha_{2}^{*}}{d\alpha_{1}}<0$, we can show reaction curves for the renewable percentage requirements set in country 1 and country 2 in an $\alpha_{1}$ and $\alpha_{2}$ space (Figure 1).

The intersection of the two reaction curves is the Cournot–Nash Equilibrium point, which is one country’s optimal response to the other country’s green quota. The slope of the reaction curves depends on the mixed partial of welfare with respect to the two choice variables, and the partial of welfare with respect to $\alpha_{2}$ as well. Since $\frac{d\alpha_{1}^{*}}{d\alpha_{2}}<0$ and $\frac{d\alpha_{2}^{*}}{d\alpha_{1}}<0$, we have a case of strategic substitutes. This implies that in a Cournot–Nash equilibrium, if one country strictly enforces its environmental target and increases its percentage requirement, in response, the other country will strategically reduce its renewable quota in order to maximize its welfare.

#### 4.1.2. Iso-Welfare Curves for Each Country

We consider a particular bundle, $\left(\alpha_{1}^{0},\;\alpha_{2}^{0}\right)$, for the two countries’ percentage requirement setting. Let $W_{1}(\alpha_{1},\;\alpha_{2})=K=W_{1}(\alpha_{1}^{0},\;\alpha_{2}^{0})$, where K is a constant. Taking the total derivative of $W_{1}(\alpha_{1},\;\alpha_{2})$ about the point $\left(\alpha_{1}^{0},\;\alpha_{2}^{0}\right)$, then we have $dK=dW_{1}(\alpha_{1}^{0},\;\alpha_{2}^{0})=\frac{\partial W_{1}}{\partial \alpha_{1}^{0}}\cdot d\alpha_{1}+\frac{\partial W_{1}}{\partial \alpha_{2}^{0}}\cdot d\alpha_{2}$, and then

(12) $$\frac{dK}{d\alpha_{1}}=\frac{dW_{1}(\alpha_{1}^{0},\;\alpha_{2}^{0})}{d\alpha_{1}}=\frac{\partial W_{1}}{\partial \alpha_{1}^{0}}+\frac{\partial W_{1}}{\partial \alpha_{2}^{0}}\cdot \frac{d\alpha_{2}}{d\alpha_{1}}$$

Along country 1’s iso-welfare curve, if the value of $\alpha_{1}$ is changed by $d\alpha_{1}$, without moving off the iso-$W_{1}$ curve, the value of $\alpha_{2}$ must also be changed by $d\alpha_{2}$ such that there is no change in $W_{1}$. So $\frac{dK}{d\alpha_{1}}=\frac{dW_{1}(\alpha_{1}^{0},\;\alpha_{2}^{0})}{d\alpha_{1}}=0$. Combined with Equation (12), we can get:

(13) $$\frac{d\alpha_{2}}{d\alpha_{1}}=-\frac{\frac{\partial W_{1}}{\partial \alpha_{1}^{0}}}{\frac{\partial W_{1}}{\partial \alpha_{2}^{0}}}$$

**Figure 2:**
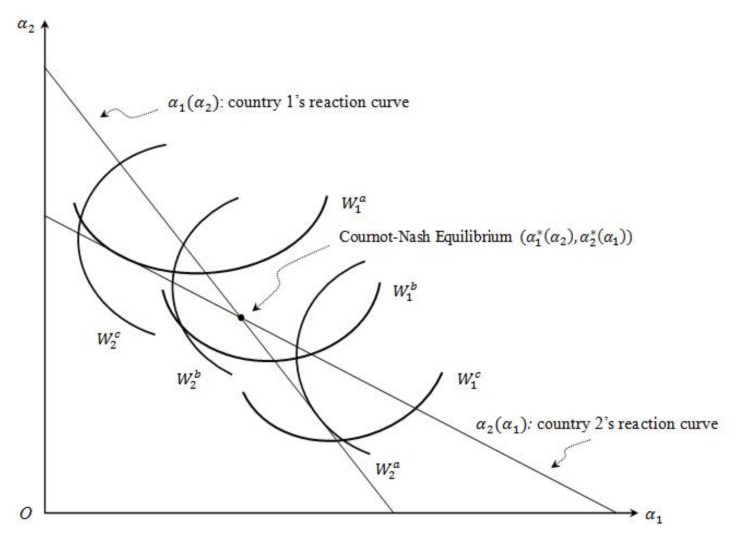
Each country’s iso-welfare curves.

Thus, the ratio of $W_{1}$’s partial with respect to $\alpha_{1}$ and $\alpha_{2}$ gives the slope of the iso-welfare curve for country 1 at point $\left(\alpha_{1}^{0},\;\alpha_{2}^{0}\right)$. We have known that $\frac{\partial W_{1}}{\partial \alpha_{2}}>0$, thus when $\alpha_{1}\in [0,\;\alpha_{1}^{*}]$, $\frac{\partial W_{1}}{\partial \alpha_{1}}\geq 0$ and $\frac{d\alpha_{2}}{d\alpha_{1}}\leq 0$; when $\alpha_{1}\in [\alpha_{1}^{*},\;1]$, $\frac{\partial W_{1}}{\partial \alpha_{1}}<0$ and $\frac{d\alpha_{2}}{d\alpha_{1}}>0$. Similarly, we can show that when $\alpha_{2}\in [0,\;\alpha_{2}^{*}]$, $\frac{\partial W_{2}}{\partial \alpha_{2}}\geq 0$ and $\frac{d\alpha_{1}}{d\alpha_{2}}\leq 0$; when $\alpha_{2}\in [\alpha_{2}^{*},\;1]$, $\frac{\partial W_{2}}{\partial \alpha_{2}}<0$ and $\frac{d\alpha_{1}}{d\alpha_{2}}>0$. The iso-welfare curves for country 1 and country 2 in the $\alpha_{1}$ and $\alpha_{2}$ space are shown below (Figure 2). In Figure 2, the lower iso-$W_{1}$ lines are associated with lower levels of welfare in country 1, so $W_{1}^{a}>W_{1}^{b}>W_{1}^{c}$.

### 4.2. Stackelberg-Type Competition

#### 4.2.1. Stackelberg Equilibrium under Full Separation

Alternatively, in a Stackelberg-type competition, the game consists of a leader and a follower. The leader wants to select its own renewable quota on the follower’s reaction curve where the leader has the highest possible welfare. Let country 1 represent the leader and country 2 represent the follower. We assume there is perfect information in this game. The game is solved with backward induction: (i) the leader considers what the best response of the follower is, that is, how the follower will respond once it has observed the renewable quota of the leader. (ii) The leader then picks a renewable quota that maximizes its welfare, anticipating the predicted response of the follower. (iii) The follower actually observes this and in equilibrium, picks the expected renewable quota as a response. It goes through the following steps:

First, to calculate the sub-game perfect Nash Equilibrium, the best response functions of the follower must be calculated. The welfare of country 2 is $W_{2}=U_{2}(x_{2}(\alpha_{2})+y_{2}(\alpha_{2}))-C_{2x}(x_{2}(\alpha_{2}))-C_{2y}(y_{2}(\alpha_{2}))-D_{2}(y_{1}(\alpha_{1})+y_{2}(\alpha_{2}))$ The best response is to find the value of $\alpha_{2}$ that maximizes $W_{2}$, given $\alpha_{1}$. Take the first-order condition of $W_{2}$ with respect to $\alpha_{2}$: $U_{2}^{\prime }\cdot (\frac{\partial x_{2}}{\partial \alpha_{2}}+\frac{\partial y_{2}}{\partial \alpha_{2}})-C_{2x}^{\prime }\cdot \frac{\partial x_{2}}{\partial \alpha_{2}}-C_{2y}^{\prime }\cdot \frac{\partial y_{2}}{\partial \alpha_{2}}-D_{2}^{\prime }\cdot \frac{\partial y_{2}}{\partial \alpha_{2}}=0$ The value of $\alpha_{2}$ that satisfies this equilibrium is country 2’s best response, and $\alpha_{2}$ is a function of the leader’s $\alpha_{1}$.

Now, the leader considers its best response function, which is calculated by considering the follower’s $\alpha_{2}$ as a function of the leader’s $\alpha_{1}$. Country 1’s welfare is given by $W_{1}=U_{1}(x_{1}(\alpha_{1})+y_{1}(\alpha_{1}))-C_{1x}(x_{1}(\alpha_{1}))-C_{1y}(y_{1}(\alpha_{1}))-D_{1}(y_{1}(\alpha_{1})+y_{2}(\alpha_{2}(\alpha_{1})))$.

The best response is to find the value of $\alpha_{1}$, say $\alpha_{1}^{*}$, that maximizes $W_{1}$, given $\alpha_{2}(\alpha_{1})$. That is, given the best response function of country 2, the renewable quota that maximizes country 1’s welfare is found. Take the first-order condition of $W_{1}$ with respect to $\alpha_{1}$: $U_{1}^{\prime }\cdot (\frac{\partial x_{1}}{\partial \alpha_{1}}+\frac{\partial y_{1}}{\partial \alpha_{1}})-C_{1x}^{\prime }\cdot \frac{\partial x_{1}}{\partial \alpha_{1}}-C_{1y}^{\prime }\cdot \frac{\partial y_{1}}{\partial \alpha_{1}}-D_{1}^{\prime }\cdot (\frac{\partial y_{1}}{\partial \alpha_{1}}+\frac{\partial y_{2}}{\partial \alpha_{2}}\cdot \frac{\partial \alpha_{2}}{\partial \alpha_{1}})=0$. So, the value of $\alpha_{1}$, say $\alpha_{1}^{*}$, that satisfies the above equilibrium is country 1’s best response. At $\alpha_{1}^{*}$, country 1’s welfare is maximized, with $W_{1}^{*}=W_{1}(\alpha_{1}^{*},\;\alpha_{2}(\alpha_{1}^{*}))$. 

**Figure 3:**
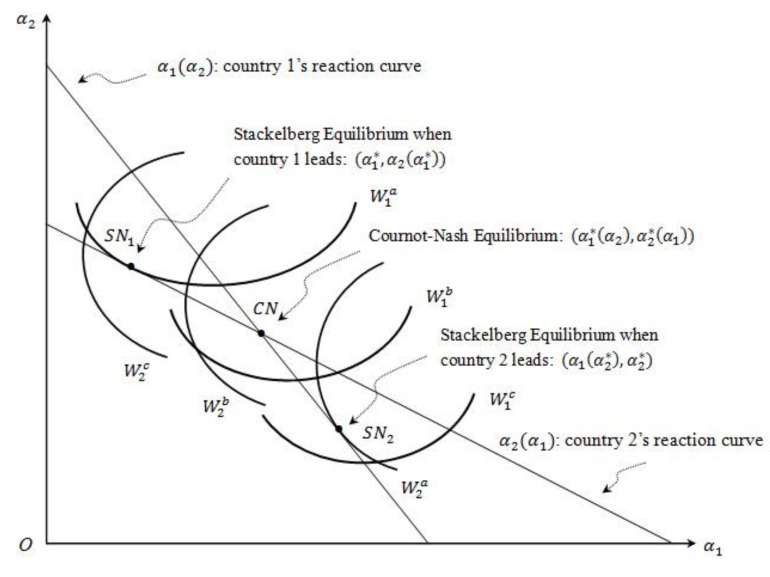
Stackelberg equilibrium.

Figure 3 shows the Cournot–Nash equilibrium, the Stackelberg equilibrium $\left(\alpha_{1}^{*},\;\alpha_{2}(\alpha_{1}^{*})\right)$ when country 1 leads, and the Stackelberg equilibrium $\left(\alpha_{1}(\alpha_{2}^{*}),\;\alpha_{2}^{*}\right)$ when country 2 leads in the $\alpha_{1}$ and $\alpha_{2}$ space. When country 1 leads, the Stackelberg equilibrium point, $SN_{1}$, locates on country 2’s reaction curve, but to the left of the Cournot–Nash equilibrium point, CN. When country 2 leads, the Stackelberg equilibrium point, $SN_{2}$, locates on country 1’s reaction line, but somewhere below the Cournot equilibrium point, CN.

#### 4.2.2. Strategic Behavior under Full Separation

Under the Stackelberg-type competition, a country would always prefer to be the leader by using an information advantage. This can be proved as follows. Let $\left(\alpha_{1}^{*},\;\alpha_{2}^{*})=(\alpha_{1}^{*},\;\alpha_{2}(\alpha_{1}^{*})\right)$ be the Stackelberg equilibrium when country 1 leads. Now, we need to show that $\alpha_{1}^{*}\leq \alpha_{1}(\alpha_{2}^{*})$, where $\alpha_{1}^{*}$ implies country 1’s choice of renewable quota when it is the leader, and $\alpha_{1}(\alpha_{2}^{*})$ implies country 1’s choice of renewable quota when it is the follower.

Suppose $\alpha_{1}^{*}>\alpha_{1}(\alpha_{2}^{*})$, applying function $\alpha_{2}(\cdot )$ to both sides of the inequality, then because $\frac{d\alpha_{2}}{d\alpha_{1}}<0$, we have $\alpha_{2}(\alpha_{1}^{*})<\alpha_{2}(\alpha_{1}(\alpha_{2}^{*}))$ or $\alpha_{2}^{*}<\alpha_{2}(\alpha_{1}(\alpha_{2}^{*}))$. Since $\frac{\partial W_{1}}{\partial \alpha_{2}}>0$, we can further get $W_{1}(\alpha_{1}(\alpha_{2}^{*}),\;\alpha_{2}^{*})<W_{1}(\alpha_{1}(\alpha_{2}^{*}),\;\alpha_{2}(\alpha_{1}(\alpha_{2}^{*})))$. Because of the definition of the reaction function, $W_{1}(\alpha_{1}^{*},\;\alpha_{2}^{*})<W_{1}(\alpha_{1}(\alpha_{2}^{*}),\;\alpha_{2}^{*})$. 

Hence, we conclude that $W_{1}(\alpha_{1}^{*},\;\alpha_{2}^{*})<W_{1}(\alpha_{1}(\alpha_{2}^{*}),\;\alpha_{2}^{*})<W_{1}(\alpha_{1}(\alpha_{2}^{*}),\;\alpha_{2}(\alpha_{1}(\alpha_{2}^{*})))$. This implies the point $\left(\alpha_{1}(\alpha_{2}^{*}),\;\alpha_{2}(\alpha_{1}(\alpha_{2}^{*}))\right)$ yields higher welfare than the point $\left(\alpha_{1}^{*},\;\alpha_{2}(\alpha_{1}^{*})\right)$, contradicting the claim that $\left(\alpha_{1}^{*},\;\alpha_{2}(\alpha_{1}^{*})\right)$ is the Stackelberg equilibrium. Therefore, the claim establishes $\alpha_{1}^{*}\leq \alpha_{1}(\alpha_{2}^{*})$. By the properties of the iso-$W_{1}$ curves, $W_{1}(\alpha_{1}^{*},\;\alpha_{2}(\alpha_{1}^{*}))>W_{1}(\alpha_{1}(\alpha_{2}^{*}),\;\alpha_{2}^{*})$. These imply that country 1 always prefers to be the leader in this game.

Then how would a country become the leader? What information advantage would it need? In the Stackelberg-type game, we solve the game with backward induction. When country 1 is the leader, country 2 wants to maximize its welfare given the leader’s $\alpha_{1}$. This is just like the Cournot condition, which gives the reaction function of country 2, $\alpha_{2}(\alpha_{1})$. Moving back to the first stage of the game, country 1 now wants to choose its $\alpha_{1}$. Looking ahead and recognizing how country 2 will respond, country 1 picks the optimal point, $\left(\alpha_{1}^{*},\;\alpha_{2}(\alpha_{1}^{*})\right)$, on country 2’s reaction curve. Therefore, to be the leader, one country needs to recognize how the other country will respond, given the former country’s own renewable quota. That is, a country has to recognize the reaction function of the other country in order to be the leader. With this information, it will be able to pick the optimal point on the other country’s reaction curve. Thus, we have the following proposition.

**Proposition** **2.** *In a Stackelberg-type competition under full separation, a country would always prefer to be the leader by using an information advantage. In order to be the leader, a country has to recognize the percentage requirement reaction function of the other country, and then it will be able to pick the optimal point on the other country’s reaction curve.*

## 5. Implications of Full Integration

In the real world, when not considering additional, nonenvironmental costs (Nonenvironmental costs include social norms, conventions, and other political reasons, and may play an important role in sustaining international environmental agreements) [20], an important incentive for the two countries with a common border to integrate their electricity markets or to self-enforce cross-border environmental agreements is that the integration or the agreements have to be profitable to both countries. In this section, we shall try to determine some implications of the impact of full integration by considering two scenarios—when the two countries are fully symmetric (symmetric electricity demands, production costs, and environmental damages) and when they are asymmetric. 

### 5.1. Fully Symmetric Scenario

**Figure 4:**
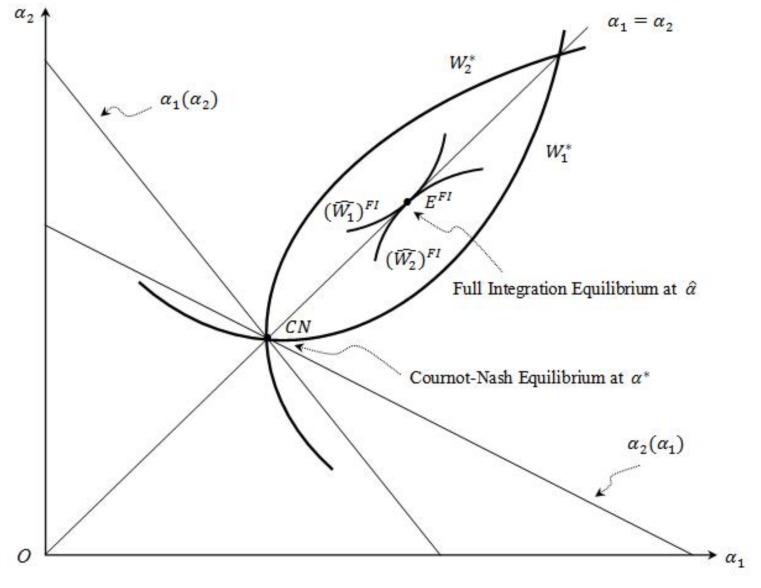
Full integration—fully symmetric scenario.

When these two countries’ utility functions, cost functions, and environmental damage functions are all symmetric, the Cournot–Nash equilibrium, $\alpha_{1}^{*}=\alpha_{2}^{*}=\alpha^{*}$, is point CN in Figure 4. Under full integration, $W_{1}^{FI}(\alpha_{1};\;\alpha_{2})+W_{2}^{FI}(\alpha_{2};\;\alpha_{1})$ is maximized subject to $\alpha_{1}=\alpha_{2}$. Since $\alpha^{*}$ is an arbitrary point on the line $\alpha_{1}=\alpha_{2}$ and $\hat{\alpha }$ maximizes $W_{1}^{FI}(\alpha )+W_{2}^{FI}(\alpha )$ at the equilibrium of full integration (point $E^{FI}$ in Figure 4), ${\left({\hat{W}}_{2}\right)}^{FI}\geq W_{2}^{*}$ and ${\left({\hat{W}}_{1}\right)}^{FI}\geq W_{1}^{*}$. Thus, as Figure 4 reveals, under full symmetry, both countries are better off and no side-payments are necessary when their electricity markets and TGC markets are fully integrated.

### 5.2. Asymmetric Scenario

When the countries’ utility functions, cost functions, and environmental damage functions are not fully symmetric, the $\alpha_{1}=\alpha_{2}$ line in Figure 4 may not intersect with the area encircled by $W_{1}^{*}$ and $W_{2}^{*}$ in the shape of a “football”. In this case, under full integration, the total welfare increases, but one country is worse off. Moreover, when side-payments between the countries are possible, $\hat{\alpha }$ at the equilibrium of full integration may still be attainable. For example, suppose $W_{1}^{*}=10$ and $W_{2}^{*}=11$ at the Nash equilibrium $\left(\alpha_{1}^{*},\;\alpha_{2}^{*}\right)$. Also, suppose that under full integration, ${\left({\hat{W}}_{1}\right)}^{FI}=15$, ${\left({\hat{W}}_{2}\right)}^{FI}=9$. The total welfare goes up, but country 2 is hurt. Suppose country 1 pays country 2 $3. Then ${\left({\hat{W}}_{1}\right)}^{FI}=12>10$ and ${\left({\hat{W}}_{2}\right)}^{FI}=12>11$. So, both countries are better off when compensation is possible between them.

Moreover, when the countries are asymmetrical, full integration could hurt the country that imports green electricity if side-payments between them are not allowed. Indeed, the full harmonization of the renewables policy eliminates the strategic behavior, and the renewable percentage requirement is raised, which, in turn, drives up the price of green electricity. Consequently, the terms of trade of the green electricity importing country deteriorate, and this damage could outweigh the benefit of trade and the profit derived from reducing pollution for the integration to harm that country.

We therefore conclude that under ideal conditions, full integration between the two countries’ electricity markets with a common TGC system would be a Kaldor–Hicks improvement if one country that is made better off could compensate the country that is made worse off, as long as the possibility for compensation exists, and thus, it does not necessarily make each country better off. Being different from a Pareto improvement where no one is made worse off, the Kaldor–Hicks improvement implies that a Pareto improving outcome can be reached by allowing those made better off to sufficiently compensate those made worse off. In our case, the Kaldor–Hicks improvement achieved under full integration may not be a Pareto improvement, since it may not benefit each country when not considering transfers between the countries and when the total emissions could be higher in a situation in which the signatory countries commit themselves to giving transfers to a third free-riding country.

### 5.3. Illustrative Example and Supportive Evidence

We now provide a numerical example to illustrate our analysis above. Assume that $U_{1}(z_{1})=A_{1}z_{1}-\frac{z_{1}^{2}}{2}$, $U_{2}(z_{2})=A_{2}z_{2}-\frac{z_{2}^{2}}{2}$, where $A_{1},\;A_{2}>0$. In addition, assume that $C_{1x}(x_{1})=\frac{a_{1}x_{1}^{2}}{2}$, $C_{2x}(x_{2})=\frac{a_{2}x_{2}^{2}}{2}$, $C_{1y}=\frac{b_{1}y_{1}^{2}}{2}$, $C_{2y}=\frac{b_{2}y_{2}^{2}}{2}$, and $D={\left(\theta_{1}y_{1}+\theta_{2}y_{2}\right)}^{2}$, where $a_{1}$, $a_{2}$, $b_{1}$, $b_{2}>0$, and $\theta_{1}$, $\theta_{2}>0$ are parameters reflecting the emissions intensities in country 1 and country 2. Following the numerical examples in Currier et al. (2012), Currier and Sun (2014), and Currier (2015), we first assume the following parameter values: $A_{1}=25$, $A_{2}=32$, $a_{1}=15$, $a_{2}=18$, $b_{1}=9$, $b_{2}=7$ and $\theta_{1}=\frac{7}{11}$, $\theta_{2}=\frac{8}{11}$. The asymmetric cost functions for these two countries reflect that country 1 has the comparative advantage in producing renewable output (green electricity exporter), and black production in country 2 is comparatively cheaper (green electricity importer). In addition, green production costs more than black production.

We compare the performance of the optimal renewables policy for each country under full integration and under full separation in Table 1. The comparison results include calculations for environmental damage, each country’s welfare, green output, black output, consumption, and imports/exports under each scenario.

We also consider the situation in which these two countries’ utility functions and cost functions are symmetric, that is, $A_{1}=A_{2}$, $a_{1}=a_{2}$, $b_{1}=b_{2}$. The parameter values are assumed as following: $A_{1}=A_{2}=32$, $a_{1}=a_{2}=18$, $b_{1}=b_{2}=7$ and $\theta_{1}=\frac{7}{11}$, $\theta_{2}=\frac{8}{11}$. The performance when these two countries’ utility functions, cost functions, and environmental damage functions are all symmetric, where $A_{1}=A_{2}=32$, $a_{1}=a_{2}=18$, $b_{1}=b_{2}=7$ and $\theta_{1}=\theta_{2}=\frac{8}{11}$ is also considered. The comparison results are shown in Table 2.

From Table 1 and Table 2, we find that full integration between the two countries’ electricity markets with a common TGC system would be a Kaldor–Hicks improvement outcome, since it may not benefit each country when not considering transfers between the countries and when the total emissions could be higher in a situation in which the signatory countries commit themselves to giving transfers to a third free-riding country.

In fact, our discussion shows the potential of a Kaldor–Hicks improvement by developing a similar system between two countries with a common well-functioning TGC market. In practice, a typical example is the “statistical transfers” system stated by the Renewable Energy Directive in the EU [2], which allows the member states to meet their national renewable targets by financing renewable energy production in other countries.

## 6. Conclusions and Policy Implications

In this paper, we investigate the performance of the optimal renewables policy under full separation and full integration scenarios for two countries’ electricity markets operated under TGC systems using a stylized model. Considering each country’s percentage requirement as a strategic choice variable, our analysis suggests that in a Cournot–Nash equilibrium under full separation, one country’s optimal renewable quota that maximizes its own welfare is strategically substitutional for the other country’s, with the existence of cross-border pollution between them. By using an information advantage of the other country’s response function, a country would always prefer to be a Stackelberg leader in the game.

We further demonstrate the possibility that a potentially fully integrated electricity market regulated by a TGC system may lead to higher welfare for each country than fully separated electricity markets with TGCs. By looking at the asymmetric cost and utility functions between the two countries and considering each country’s comparative advantage in producing green/black electricity, we find that the optimal renewables policy generally performs better under full integration than under full separation in terms of welfare, environmental damage, and black output levels, when the possibility of transfers between the countries exists. Though country 2’s green output level does not change much, this may be due to its comparative disadvantage in producing renewable electricity compared with country 1. We then look at the symmetric cost and utility functions for the countries and find results consistent with the asymmetric case. Thus, full integration between the two countries’ electricity markets with a common green certificate system can be a Kaldor–Hicks improvement, as long as the possibility of compensation exists. This result is significant in view of the fact that even though the debate about the full harmonization or full independence of renewable electricity support persists, an immediate demand for a convergence of national renewables policy schemes has been seen in the European Union [9], and *statistical transfers* have been allowed by the EU Renewable Energy Directive [2].

Prospects where two or more countries cooperate on a cross-border policy scheme have been the subject of more discussion in recent years. Some early cooperations between countries have been gradually implemented, for instance, between Norway and Sweden [9,11,12]. A couple of studies in literature have provided empirical evidence for our theoretical results. For example, Verhaegen et al. [34] evaluated the possibility of a single European certificate system to promote renewables. They applied both feed-in tariffs and TGCs combined with quota obligations and demonstrated the need to expand borders of a green certificate system. However, the EU’s desire for a European-wide harmonized support scheme is contrasted with the situation in Belgium. Wedzik et al. [35] suggested that although FIT have outperformed the TGC-based systems in EU on a national level, this could be reversed on an EU-wide level. It could be very difficult to introduce EU uniformed FIT, while TGC systems might benefit from a broad pan-EU market, making a fulfillment of renewable energy sources (RES) requirements easier for some member states due to geographical factors. Chen et al. [36] applied a stochastic robust optimization method to the Beijing–Tianjin–Hebei region in China and found that a multi-region TGC mechanism is a cost-effective pathway to cope with carbon reduction and can greatly alleviate financial pressure on the government to provide renewable energy subsidies. Further, Aune et al. [10] numerically showed that allowing for full trade in green certificates and imposing a common renewable target for countries can ensure cost-reducing potential in achieving the renewable target.

While the debate between the supporters of full harmonization and the supporters of full national independence of renewable-promoting policies continues, the EU government has seen an urgent demand for a convergence of national policy schemes. They note that “a greater convergence of national support schemes to facilitate trade and move towards a more pan-European approach to development of renewable energy sources must be pursued” [9,13]. Our model is static in nature, assuming the partial equilibria of electricity and TGC markets. It highlights the demand for cost and benefit studies in electricity markets for the development of renewable energy, since renewables are expensive on a direct cost basis, with many additional unaccountable benefits and costs. In fact, some renewable sources heavily depend on the local weather, for example, solar and wind, and thus, they are intermittent and the least controllable. The power from these resources needs to be evaluated based on the time at which it is produced. The functioning of a dynamic TGC market can be investigated by experimental simulation models. Ford et al. [37] experimentally investigated the functioning of a TGC market in a dynamic context. They suggested that dynamics in TGC markets are likely to be complicated due to formulation of expectations, delays in possession of capacity, and the likelihood of storing green certificates, and so forth. This may be particularly true for power systems based on solar and wind [11]. Hence, based on the case of the Swedish–Norwegian electricity certificate market, Hustveit et al. [38] suggested that regulatory changes should be implemented carefully to avoid increased uncertainty and a consequent increase in price volatility. In addition, adjusting for both the market value of the electricity generated and the associated environmental and nonenvironmental externalities is useful for governments to implement reasoned renewables support schemes [39].

In practice, electricity markets are currently fairly concentrated, and it remains a great challenge to determine the socially optimal percentage requirements due to the limited information that the regulator has about production costs and consumers’ demand. Currier [40] devises a branch and bound regulatory adjustment process to determine the optimal renewable quota iteratively within the context of Cournot competition. Clearly, a more general analysis could investigate the determination of the socially optimal percentage requirement under multiple market structures between countries.

For a common electricity market to work, appropriate interconnection agreements and the transmission infrastructure connectivity between countries are basic conditions for markets’ integration. In addition, an international TGC system requires co-existing national support schemes, such as feed-in tariffs and investment aid, for electricity from renewable sources to be harmonized across countries in order to make it more compatible with an integrated electricity market [7,23]. Therefore, restrictions in the transmission capacity under free trade between countries remains a problem. On the other side, due to each country’s welfare incentive to “cheat” on the agreement, the welfare maximum under full integration may not be sustainable. In such cases, various cost reduction incentives at each equilibrium under full integration may be investigated, and a more general model that allows for the presence of market power in both the electricity market and the TGC market may be studied. These and other related questions we hope to address in future research.

## Figures and Tables

**Table 1:** Comparison results for full integration and full separation (asymmetric functions).

	Full Integration	Full Separation
	$\alpha^{*}=0.4534$	${\left(\alpha_{1}^{*},\;\alpha_{2}^{*}\right)}^{CN}=(0.4451,\;0.3356)$
$W_{1}$	41.6739	31.6214
$W_{2}$	71.1656	68.9751
D	10.1703	14.7239
$x_{1}$	2.0987	1.6502
$x_{2}$	1.7489	1.7554
$y_{1}$	2.0294	2.0575
$y_{2}$	2.6093	3.4758
$z_{1}$	0.7432	3.7076
$z_{2}$	7.7432	5.2312
Imports/Exports	3.3850	0
p	18.2648	$\left(p_{1}^{FS},\;p_{2}^{FS})=(18.5171,\;24.3309\right)$
$p_{c}$	13.2159	$\left(p_{c_{1}}^{FS},\;p_{c_{2}}^{FS})=(6.2356,\;7.2653\right)$
$p+p_{c}$	31.4807	$\left(p_{1}^{FS}+p_{c_{1}}^{FS},\;p_{2}^{FS}+p_{c_{2}}^{FS})=(24.7526,\;31.5962\right)$
$p+\alpha p_{c}$	24.2568	$\left(p_{1}^{FS}+\alpha_{1}p_{c_{1}}^{FS},\;p_{2}^{FS}+\alpha_{2}p_{c_{2}}^{FS})=(21.2924,\;26.7688\right)$

Note: subscript “FS” refers to full separation, and “CN” refers to Cournot–Nash equilibrium.

**Table 2:** Comparison results for full integration and full separation (symmetric functions).

	$A_{1}=A_{2}$ $,\;a_{1}=a_{2}$ $,\;b_{1}=b_{2}$		$A_{1}=A_{2}$ $,\;a_{1}=a_{2}$ $,\;b_{1}=b_{2}$ $,\;\theta_{1}=\theta_{2}$	
	FI	FS	FI	FS
	$\alpha^{*}=0.4199$	${\left(\alpha_{1}^{*},\;\alpha_{2}^{*}\right)}^{CN}=(0.3398,\;0.3516)$	$\alpha^{*}=0.4385$	$\alpha_{1}^{*}=\alpha_{2}^{*}=0.3561$
$W_{1}$	63.3854	62.0049	61.4217	59.3002
$W_{2}$	63.3854	61.4797	61.4217	59.3002
D	15.0281	21.5262	15.3657	23.4849
$x_{1}$	2.0580	1.7741	2.1043	1.8424
$x_{2}$	2.0580	1.8242	2.1043	1.8424
$y_{1}$	2.8429	3.4466	2.6949	3.3317
$y_{2}$	2.8429	3.3637	2.6949	3.3317
$z_{1}$	4.9009	5.2207	4.7992	5.1741
$z_{2}$	4.9009	5.1879	4.7992	5.1741
Imports/Exports	0	0	0	0
p	19.9	(24.1263, 23.546)	18.8646	(23.322, 23.322)
$p_{c}$	17.1439	(7.8072, 9.2889)	19.0124	(9.8405, 9.8405)
$p+p_{c}$	37.0439	(31.9335, 32.8348)	37.877	(33.1625, 33.1625)
$p+\alpha p_{c}$	27.0992	(26.7793, 26.8121)	27.2008	(26.8259, 26.8259)
